# Dose optimization of β-lactams antibiotics in pediatrics and adults: A systematic review

**DOI:** 10.3389/fphar.2022.964005

**Published:** 2022-09-21

**Authors:** Abdul Haseeb, Hani Saleh Faidah, Saleh Alghamdi, Amal F. Alotaibi, Mahmoud Essam Elrggal, Ahmad J. Mahrous, Safa S. Almarzoky Abuhussain, Najla A. Obaid, Manal Algethamy, Abdullmoin AlQarni, Asim A. Khogeer, Zikria Saleem, Muhammad Shahid Iqbal, Sami S. Ashgar, Rozan Mohammad Radwan, Alaa Mutlaq, Nayyra Fatani, Aziz Sheikh

**Affiliations:** ^1^ Department of Clinical Pharmacy, College of Pharmacy, Umm Al-Qura University, Makkah, Saudi Arabia; ^2^ Department of Microbiology, Faculty of Medicine, Umm Al-Qura University, Makkah, Saudi Arabia; ^3^ Department of Clinical Pharmacy, Faculty of Clinical Pharmacy, Al Baha University, Al Baha, Saudi Arabia; ^4^ Department of Pharmaceutics, College of Pharmacy, Umm Al-Qura University, Makkah, Saudi Arabia; ^5^ Department of Infection Prevention and Control Program, Alnoor Specialist Hospital, Makkah, Saudi Arabia; ^6^ Infectious Diseases Department, Alnoor Specialist Hospital, Makkah, Saudi Arabia; ^7^ Plan and Research Department, General Directorate of Health Affairs of Makkah Region, Ministry of Health, Makkah, Saudi Arabia; ^8^ Medical Genetics Unit, Maternity and Children Hospital, Makkah Healthcare Cluster, Ministry of Health, Makkah, Saudi Arabia; ^9^ Department of Pharmacy Practice, Faculty of Pharmacy, Bahauddin Zakariya Univrsity, Multan, Pakistan; ^10^ Department of Clinical Pharmacy, College of Pharmacy, Prince Sattam Bin Abdulaziz University, Al-Kharj, Saudi Arabia; ^11^ Pharmaceutical Care Department, Alnoor Specialist Hospital, Ministry of Health, Makkah, Saudi Arabia; ^12^ General Department of Pharmaceutical Care, Ministry of Health, Riyadh, Saudi Arabia; ^13^ King Abdulaziz University, Jeddah, Saudi Arabia; ^14^ Usher Institute, The University of Edinburgh, Edinburgh, United Kingdom

**Keywords:** dose optimization, beta-Lactams, carbapenems, penicillins, cephalosporins

## Abstract

**Background:** β-lactams remain the cornerstone of the empirical therapy to treat various bacterial infections. This systematic review aimed to analyze the data describing the dosing regimen of β-lactams.

**Methods:** Systematic scientific and grey literature was performed in accordance with Preferred Items for Systematic Reviews and Meta-Analysis (PRISMA) guidelines. The studies were retrieved and screened on the basis of pre-defined exclusion and inclusion criteria. The cohort studies, randomized controlled trials (RCT) and case reports that reported the dosing schedule of β-lactams are included in this study.

**Results:** A total of 52 studies met the inclusion criteria, of which 40 were cohort studies, 2 were case reports and 10 were RCTs. The majority of the studies (34/52) studied the pharmacokinetic (PK) parameters of a drug. A total of 20 studies proposed dosing schedule in pediatrics while 32 studies proposed dosing regimen among adults. Piperacillin (12/52) and Meropenem (11/52) were the most commonly used β-lactams used in hospitalized patients. As per available evidence, continuous infusion is considered as the most appropriate mode of administration to optimize the safety and efficacy of the treatment and improve the clinical outcomes.

**Conclusion:** Appropriate antibiotic therapy is challenging due to pathophysiological changes among different age groups. The optimization of pharmacokinetic/pharmacodynamic parameters is useful to support alternative dosing regimens such as an increase in dosing interval, continuous infusion, and increased bolus doses.

## Introduction

In the past few years, increasing trend of antibiotic resistance challenges the efficacy of currently available antibiotics. It is because of the global dissemination of multi-drug resistant (MDR) microorganisms causing more than 23,000 death annually in the United States (Pacios et al., 2020). The higher mortality rates associated with methicillin-resistant Staphylococcus aureus (MRSA) were observed in East Africa (Wangai et al., 2019). A study reported that about 96,000 patients were died due to MDR infection in Southern Asia (Khan et al., 2016). Similarly, the morbidity rates associated with MDR are also high, particularly in low- and middle-income countries (LMICs) due to lack of resources, inadequate microbiological testing methods and treatment interventions (Atif et al., 2020). According to the Centers of Disease Control and Prevention (CDC), the economic burden associated with drug-resistant infections estimated US$3.5 billion annually (Caron et al., 2010). One of the major causes for the spread of these infections is the injudicious use of antibiotics. The injudicious use of antibiotics can contribute to increased mortality, morbidity, and overall healthcare costs (Lesprit and Brun-Buisson, 2008). The use of unnecessarily broad-spectrum antibiotics is common in empirical as well as targeted therapy (Mettler et al., 2007). Many healthcare professionals have limited knowledge regarding antibiotic use and resistance and do not follow guidelines. Expert-based strategies and policies regarding the initiation and implementation of an antibiotic stewardship program (ASP) are recommended by different organizations such as World Health Organization (WHO), CDC, and Infectious Diseases Society of America (IDSA) (van Limburg et al., 2014; Gross et al., 2019).

Antibiotic stewardship program (ASP) is one of the main effective approaches to promote the rational use of antibiotics and combat antibiotic resistance. ASP also helps to optimize the treatment of infectious diseases, improve prescribing behavior, ensure cost-effective therapy, minimize the side effects related to antibiotic use, including resistance (Pollack and Srinivasan, 2014). Lee and his colleagues implemented ASP in children’s hospital that results in the reduction of antibiotic acquisition costs of about US$200,000 (Lee et al., 2017). Data published on ASP in intensive care units have demonstrated significant improvement in antibiotic consumptions (Kaki et al., 2011; Haseeb et al., 2020; Haseeb et al., 2021a; Alghamdi et al., 2021). To optimize the antibiotic use, many strategies in ASP intervention including identification of patient with bacterial infection, appropriate selection of treatment using pharmacokinetics-pharmacodynamic (PK-PD) characteristic to optimize the antibiotic dosing and modalities, de-escalation of antibiotics and shortening of therapy duration were employed (Luyt et al., 2014).

Dose optimization includes optimization of antibiotic dosing based on patient characteristics (e.g., weight, age, renal/liver function), PK-PD parameters of the drug (e.g., concentration or time-dependent activity), and causative microorganisms (Haseeb et al., 2021b; Haseeb et al., 2022). An appropriate dosing is the mainstay of antibiotic therapy, which intensifies the PK and PD profiles of drugs and has a huge impact on therapeutic outcomes, dose-dependent toxicity as well as the emergence of antibiotic resistance (He et al., 2020). For instance, administering single dose of aminoglycosides instead of multiple doses not only improve bacterial eradication but also reduces the risk of ototoxicity and nephrotoxicity. The continuous infusion or prolonged/extended infusion of β-lactams instead of administering bolus is recommended as an advanced dose optimization strategy. This strategy not only improves therapeutic outcomes but also reduce the mortality rates for all patients infected with resistant pathogens. Multisite studies reported that for some particular antibiotics, PD profiles can be assessed to improve the efficacy by changing the mode of administration (Felton et al., 2012; Georges et al., 2012; Falagas et al., 2013). The awareness regarding how dosing strategies are employed is needed for the selection of appropriate antibiotics (Roberts et al., 2014).

The selection of dosing regimen for antibiotics are usually based on summary endpoints such as PK/PD indices and point estimates of effect in terms of MIC. Multisite site studies documented that antibiotics have been categorized in accordance with the relationship between effect and three PK/PD indices: 1) the ratio of the maximal unbound (free) drug concentration to MIC (*f*Cmac/MIC), 2) the ratio of area under the drug-concentration-time curve to the MIC (*f*AUC/MIC), or 3) the percentage of a 24-h time interval that unbound drug concentration exceeds to MIC (*f*t>MIC) (Andes and Craig, 2002; MacGowan and Bowker, 2002; Nielsen et al., 2011). These indices are commonly utilized as targets in the dose selection process. In Monte Carlo simulations, between-patient variability in PK.PD parameters is considered and the probability of the target attainment (PTA) is estimated on the basis of stochastic simulations from the model (Mouton et al., 2004; Owens Jr et al., 2005). On the basis of existing literature, the activities of β-lactams antibiotics have been categorized as being dependent on the *f*T>MIC (Leggett et al., 1989; Gustafsson et al., 2001).

β-lactams (penicillins, cephalosporins and carbapenems) are broad-spectrum antibiotics that are used widely to treat various bacterial infections in healthcare settings particularly in intensive care units (ICUs) and may be targeted by antibiotic stewardship initiatives (Masich et al., 2018b). Optimal treatment needs appropriate dosage, modes of administration and dosing schedules (Delattre et al., 2017). The clinician’s knowledge concerning dose optimization of β-lactams is broadened but still faces some issues in implementing dosing-based approaches (Grupper et al., 2016). The initiation and implementation ASP and guidelines for β-lactams can improve the clinical outcomes and decrease the spread or emergence of antibiotic resistance (Hammond et al., 2019). Therefore, optimization of antibiotic therapy is an important consideration for clinician worldwide (Cotta et al., 2015). This systematic review was aimed to assess the data describing the dose optimization of β-lactams.

## Materials and methods

### Data sources and searches

We performed systematic scientific and grey literature search according to Preferred Items for Systematic Reviews and Meta-Analysis (PRISMA) guidelines from October 2021 to January 2022 (Moher et al., 2015). Two independent approaches were followed. Comprehensive grey literature and peer-reviewed literature were performed independently by two reviewers. The reference lists of the relevant articles and related reviews were also searched manually for additional studies. Complementary research was also performed to identify the most recent studies. The search items included “antibiotic” or “dose optimization” or “pharmacokinetic” or “pharmacodynamic” or “drug administration” or “β-lactams” or “penicillins” or “cephalosporins” or “carbapenems” or “ampicillin,” “amoxicillin,” or “piperacillin,” or “ceftriaxone” or “cefuroxime” or “cefixime” or “ceftaroline” or “ceftazidime” or “meropenem” or imipenem” or “doripenem” or “‘aztreonam.”

### Inclusion and exclusion criteria

All the studies found were reviewed for eligibility. The studies retrieved from the aforementioned search strategies were combined and duplicates were removed. Full-text articles on dose optimization of antibiotics were included in this review. The inclusion criteria include articles written in English and published in peer-reviewed journals. Articles published after 2000 were included in this review to ensure the current dosing recommendation. However, the exclusion criteria were review articles, letters to editor, animal studies, no full-text availability, conference abstracts, and *in vitro* studies. Two reviewers screened titles and abstracts as per eligibility criteria to identify potential publications independently at first. Then full-text was assessed for final inclusion. The disagreements were resolved by discussion between 2 reviewers or by consulting third reviewers. The type of studies included were cohort study, case reports and randomized controlled trial (RCT).

### Quality assessment

The quality assessment was carried out using New Castle-Ottawa Scale (NOS) scale for cohort studies and Cochrane bias tool for randomized controlled trials. The NOS scale categorizes the data into three subscales, i.e., selection, comparability and outcomes (Wells et al., 2014). However, the Cochrane assessment tool validates the randomized controlled studies (RCT) by assessing the risk of bias in each study (Higgins et al., 2019). This tool is structured into domains (random sequence generation, allocation concealment, blinding of patients and personnel, blinding of outcome assessment, incomplete outcome data and other bias) through which bias of each included study might be introduced in the results. The judgment is generally based on “high risk,” “low risk” and “unclear.” Each article was independently assessed by two experts. Reviewers compared their results and differences were then sorted by discussion.

### Data extraction

The data was extracted from text, table and graph from each included study and was recorded in the pre-specified data collection form. This customized data form includes the following information; study characteristics (author’s name, year of publication, design, and sample size), patient characteristics (patient clinical condition, prescribed antibiotics, dosing regimen, outcomes of interests, and dosing recommendation). Data extraction was completed by one reviewer and it was then reviewed by another reviewer. Disagreements were addressed by discussion between two reviewers or consultation with the third reviewer if necessary.

## Results

### Characteristics of selected studies

**FIGURE 1:**
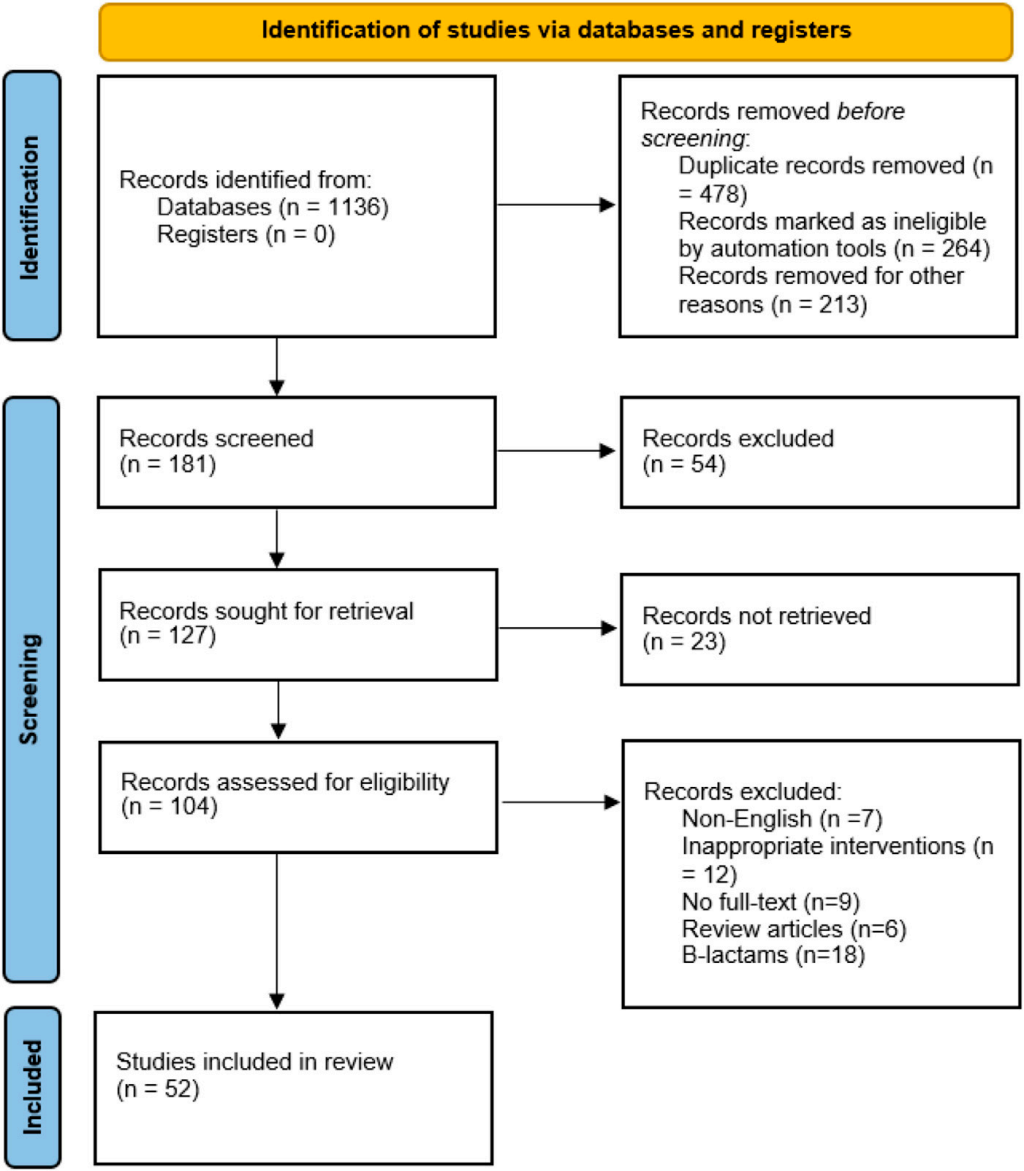
PRISMA flow diagram.

Of the 1,136 relevant published articles identified, 181 articles were initially proved eligible after duplicates were removed and abstracts screened. Various articles were retrieved from reference lists of the selected studies, other systematic reviews, and personal files. Majority of the studies were excluded some are Monte Carlo simulation studies where there were no patients involved. Of 127 articles, the data were not retrieved from 23 articles, therefore, excluded. After screening of articles, 104 articles met the eligibility criteria. A total of 52 studies were excluded due to following reasons: inappropriate intervention (*N* = 12), literature reviews (*N* = 6), non-English (*N* = 7), no full-text available (*N* = 9), and non-β-lactams (*N* = 18). The 52 articles met the inclusion criteria for this systematic review. The PRISMA flow diagram for studies selection is shown in the the Figure 1. Data extraction was performed for 47 full text articles with data on β-lactams. A complete list of all 47 articles and extracted Pk-data is presented in Tables 1, 2. All the 47 articles included were published in English of which 12 were RCT and 18 were cohort studies. The quality of case reports was not assessed because no validated tool is available. Therefore, we used Joanna Briggs institute (JBI) critical checklist for case reports (Ma et al., 2020).

**TABLE 1:** Dose optimization of β-lactams among pediatrics.

Antibiotics	Author and year	Country	Study design	Sample size	Characteristics of patients	Dosing practice	Pk parameters	Patients achieving targets (PAT)/Clinical outcomes	Dosing recommendation
Penicillins									
Amoxicillin Tang et al. (2019)	Tang et al. (2019)	China	Multi-center prospective study	187	Patients with neonatal sepsis	For EOS 25 mg/kg BID intravenous bolus for over 5 min or infusion for over 30min. For LOS 25 mg/kg QID 25 mg/kg TID	For premature infants Cl (0.11 L/kg/h) for term neonates Cl (0.25 L/kg/h)	For EOS 99.0% of premature neonates and 87.3% of term neonates achieving PD targets using MIC breakpoint of 1 mg/L. For LOS 86.1% of premature neonates, 79.0% of term neonates using MIC breakpoint of 2 mg/L	To ensure efficacy and to avoid emergence of resistance, T>MIC target above 70% of dosing interval was selected as most safe endpoint
Amoxicillin Wu et al. (2021)	Wu et al. (2021)	China	Single center prospective study	47	Patients with Meningitis, sepsis, pneumonia	25 mg/kg twice a day intravenous 60 mg/kg thrice a day intravenous	V (0.25–2.58 L/kg); Cl (0.31 L/kg/h)	22.4% infants reaching PD targets using dose regimen 25 mg/kg BID and 27.9% infants using dose regimen 60 mg/kg TID	Change antibiotic for infection caused by E. coli with MIC of 8 mg/ml
Amoxicillin D’Agate et al. (2020)	D’Agate et al. (2020)	United Kingdom	Meta-analytical modeling approach	44	Patients with Neonatal sepsis	125 mg bid with patients’ weight < 4 kg 250 mg bid with patients’ weight > 4.0 kg	Weight < 4 kg Cmax (26 mg/L); Cmin (14 mg/L). Weight >4 kg Cmax (32 mg/L); Cmin (12 mg/L)	Weight < 4 kg AUC: 254 mg.h/mL T>MIC (2 mg/L): 11.9 T>MIC (4 mg/L):11.8 T>MIC (8 mg/L):11.4 Weight > 4 kg AUC: 274 mg.h/mL T>MIC (2 mg/L): 11.9 T>MIC (4 mg/L):11.9 T>MIC (8 mg/L): 11.6	Weight-banded dose regimen should be considered for neonatal sepsis
Amoxicillin + clavulanic acid De Cock et al. (2015)	De Cock et al. (2015)	Belgium	Cohort	50	Patients with mixed conditions	25–30 mg/kg every 6 h intravenously	Amoxicillin Cl (17.97 L/h/70 kg); V1 (9.07L/70 kg); V2 (5.43 L/kg); V3 (11.24 L/kg) Clavulanic acid Cl (12.20 L/h/70 kg); V (11.60 L/70 kg); V2 (9.8 L/kg)	For prophylaxis The clinical failure rate was 32%; for treatment it was 34.4%	25 mg/kg every 4 h intravenously. 1-h infusion was preferred to bolus dosing for patients with augmented renal function
Piperacillin + tazobactam Béranger et al. (2019)	Agathe Beranger et al., 2019	France	Cohort, PK population model	50	Patients with pneumonia, peritonitis, BSI, mediastinitis, UTIs, skin abscess	300 mg/kg/day, intermittent infusions every 6 h	Half-life (0.9 h); Cl (2.6 L/hr/70 kg); Vd (4.6 L/70 kg)	Extended or continuous infusions attained PK targets (50% fT [MIC or 100% fT [MIC),	Continuous or extended infusions were the most adequate administration regimens for treatment of various infection
Piperacillin + tazobactam (Cies et al., 2014)	Cies et al. (2014)	Pennsylvania	Cohort	13	Patients with febrile neutropenia, pneumonia, burn, sepsis, enterocolitis	400 mg/kg/day in 4 divided doses	Vp (0.262 + 0.177 L/kg); Vc (0.249 L/kg); Vd (0.511L/kg); Cl (0.299 L.h/kg); Half-life (1.39 + 0.62 h)	100 mg/kg every 6 h administered as a 3-h prolonged infusion achieved 77.7% PTA and 400 mg/kg administered as a 24-h continuous infusion achieved 74.8% PTA	400 mg/kg/day in 4 doses as 3-h infusion or as continuous infusion 400 mg/kg/day in continuous or extended infusions, for children with augmented renal clearance
Piperacllin tazobactum Nichols et al. (2016)	Nichols et al. (2016)	United States	Cohort	12	Patients with pneumonia, VAP, sepsis, typhlitis	100/12.5 mg/kg TID infused over 4 h	Piperacillin Cmax (11.9 + 3.63 mg/L); Cmin (15.5 + 11.0 mg/L); Cl (0.22 + 0.07 L/h/kg); Vd (0.43 + 0.16 L/kg) Tazobactum Cmax (17.6 mg/L); Cmin (2.4 + 2.0 mg/L); Cl (0.19 + 0.007 L/h/kg); Vd (0.37 + 0.14 L/kg)	All extended-infusion dose regimens achieved PTAs of > 90% at MICs of <16 mg/L. Only the 3-h infusion regimens given every 6 h achieved PTAs of > 90% at an MIC of 32 mg/L	The doses of above 80/10 mg/kg given every 8 h and infused over 4 h achieve adequate PD targets in critically pediatrics
Piperacillin De Cock et al. (2017)	De Cock et al. (2017)	Belgium	Cohort. Pharmacokinetic study	47	Patients with TRIs, GIT, burns, postoperative, oncology, neurological disorders	300 mg/kg/day in 4 doses, infusion in 5–30 min	Piperacillin Cl (0.25 L/kg/h); V1 (0.13 L/kg); V2 (0.11 L/kg) Tazobactam Cl (0.13 L/kg/h); V1 (0.13 L/kg); V2 (0.11 L/kg)	For intermittent dosing regimens the PTA was of 90% (75 mg/kg piperacillin every 4 h, infusion over 2 h; 100 mg/kg every 4 h over 1 or 2 h). For continuous dosing regimens, PTA was 100% after loading dose	A loading dose of 75 mg/kg over 1 h followed by continuous infusion 300–450 mg/day is recommended
Cephalosporins									
Cefazolin Cies et al. (2019)	Cies et al. (2019)	United States	Cohort, prospective open-label pharmacokinetic study	41	Patients with peri-operative surgical prophylaxis	25 mg/kg as a bolus over 5 min within 60 min of the first surgical incision and an additional 25 mg/kg dose to a maximum of 1,000 mg was added to the CPB priming solution	Birth–6 months CL 0.009 ml/min/kg Vd 0.598 L/kg 7 months–3 years Cl 0.01 ml/min/kg Vd 0.786 L/kg 4–16 years Cl (0.007 ml/min/kg): Vd (3.4 L/kg)	-	mixing cefazolin in the CPB circuit priming solution was effective in maintaining cefazolin serum concentrations during surgery
Cefazolin De Cock et al. (2016)	De Cock et al. (2016)	Belgium	Cohort, prospective pharmacokinetic study	56	Patients with Cardiac surgery	25 mg/kg with maximum of 2000 mg/dose, IV as a bolus, 4 doses in total before, during and after surgery	Cl (0.229 L/h/kg; V1 (0.284 L/kg); V2 (0.351 L/kg)	The study dosing regimen was between 62% and 70% achieved PD targets during surgery and 89–98%after surgery while the PTA of proposed regimen was 88–99%	The dosing regimen (40 mg/kg, 30 min before surgical incision; 20 mg/kg, at start of CPB; 20 mg/kg, at the start of rewarming on CPB; 40 mg/kg, 8 h after the third dose; 40 mg/kg 8 h after the fourth dose) was considered effective undergoing cardiac surgery
Cefotaxime Béranger et al. (2018)	A Beranger et al., 2018	France	Cohort	64	Patients with mixed conditions	100 mg/kg/day–300 mg/kg/day in 4 doses, in patients > 50 kg the adult dose of 3 d 1,000 mg was Used	Cl (14.7 L/h/kg); Vd (21.4 L); t½ (0.34–1.15 h)	The PTA was 100% using dosing regimen 100 mg/kg/day as continuous infusion	100 mg/kg/day as continuous infusion is recommended
Cefotaxime Hartman et al. (2019)	Hartman et al. (2019)	Netherland	RCT	37	Patients with Meningococcal septic shock	100–150 mg/kg/day in 3–4 doses	-	PTA ranged from 14.7% for MIC 16 mg/L to 95.6% for MIC of 0.125 mg/L	Not given
Ceftaroline Cies et al. (2018b)	Cies et al. (2018)	Pennsylvania	Cohort	7	Patient with MRSA infections	60 mg/kg/day (1 patient with 54 mg/kg/day) in 4 doses	Vd (0.17–0.84 L/kg) Cl (1.57–6.11 ml/min/kg); t½ (0.98–2 h); k (0.50.33–0.64 h)	All patients needed a dose alteration or non-standard dose to reach the target of fT > 4–6 × MIC 40%	For bloodstream infections, pneumonia, and meningitis with MRSA, dosing every 6 h is advised. For patients with increased Vd, a dose of 15 mg/kg is advised
Cefuroxime Olguin et al. (2008)	Olguin et al. (2008)	Mexico	Cohort	11	Patients with septicimeia and septic shock	100 mg/kg every 6 h by intravenous infusion for 30 min	Control Vd (1.5 L/kg), Cl (0.55 L/kg/h); AUC (116.4 μg/ml/h) severely ill Vd (1.6 l/kg); Cl (0.48 L/kg/h); AUC (121.6 µg/ml/h) very severely ill Vd (3.1 L/kg); Cl (1.87 L/kg/h); AUC (190.7 µg/ml/h)	-	Not given
Ceftraixone Fukumoto et al. (2009)	Fukumoto et al. (2009)	Japan	Cohort	21	Patients with pneumonia	50 mg/kg/day, intravenously at a constant rate about 60-min period	Cpeak (546 µg/ml); Ctrough (25.0 µg/ml); Half-life (4.87 h); Vd (0.128 L/kg); Cl (0.0179L/h/kg)	-	The administration of ceftriaxone once daily to pediatric population with pneumonia was shown to be effective bacteriologically as well and pharmacokinetically
Ceftriaxone Khan et al. (2020)	Khan et al. (2020)	China	Cohort Open-label pharmacokinetic study	99	Patients with CAP	50–100 mg/kg once a day (QD) or two times a day (BID) over 30 min as intravenous infusion	At a steady state, Cl (0.03 L/h/kg); Vd (0.16 L/kg); AUC0–24 (460.42291.3 mg*h/L)	Using 60% fT > MIC as the PD target, the PTA was 99.4% for dosing regimen 50 mg/kg QD; 51.2% for 50 mg/kg QD; 100% for 75 mg/kg BID; 68.9% for 75 mg/kg QD; 100% for 100 mg/kg BID; 81.8% for 100 mg/kg QD.	A dose regimen of 100 mg/kg every 24 h produced satisfactory target attainment rates
Carbepenems									
Imipenem Giannoni et al. (2006)	Giannoni et al. (2006)	Switzerland	Cohort	19	Patient with mixed conditions	100 mg/kg/day in 3–4 doses, q8h and q6h infusion in 30 min	after first dose: T1/2 (1.22 h ± 0.47); Cl (0.27 L/kg/h ± 0.11); Vd (0.42 L/kg ± 0.13); Vss (0.30 ± 0.1) Steady state t½ (1.35 h ± 0.38); Cl (0.34 L/kg/h ± 0.14);Vd (0.64 L/kg ± 0.3);Vss (0.46 ± 0.25)	The dose regimen (100 mg/kg/day) prescribed by the physicians ensured a ∫T>MIC of 70%–100% for all recovered pathogens except the methicillin-resistant S epidermidis isolate	the higher-range dose of 100 mg/kg/day was uniformly appropriate over the whole pediatric population tested, irrespective of the q6h or q8h administration schedule
Meropenem Cies et al. (2015)	Cies et al. (2015)	Pennsylvania	Case report	1	Patient with Ventriculitis	40 mg/kg intravenously every 6 h, infused over 30 min	Intermittent dosingCp (12 μg/ml after 2 h) Ccsf (1 μg/ml after 2 h and 0.5 μg/ml after 4 h) Continuous dosingCp (13 μg/ml); CCSF (0.5 μg/ml)	Continuous infusion gave PTA of 100%	The continuous-infusion dosing regimen allowed for 100% PTA in the serum and CSF and a successful clinical outcome
Meropenem Cies et al. (2017b)	Cies et al. (2017)	Pennsylvania	Cohort	9	mixed	40 mg/kg/day to 160 mg/kg/day over 2–4 doses, infusion in 30 min 1 patient received continuous dosing of 200 mg/kg/day 1 patient received 100 mg/kg/day in 2 doses with prolonged infusion of 4 h	Meropenem Cl: 6.99 ml/kg/min ± 2.5Vc: 0.57 L/kg ± 0.47Kcp: 2.512 h^−1^ ± 1.449Kpc: 3.268 h^−1^ ± 1.667Total Vd 0.78 L/kg ± 0.73	Target: fT > MIC 40% and 80% for MICsfrom 0.03–32 mg/LPTA of 90% defined as optimal	120–160 mg/kg/day as continuousinfusion
Meropenem Tan et al. (2018)	Tan et al. (2018)	Singapore	prospective single-center, pharmacokinetic study	9	Patients with sepsis	40 mg/kg q12 h over a 30 min infusion	CL (0.091 L/h/kg); half-life (3.9h)	32% patients achieve PD targets by using standard dose regimen (of 40 mg/kg/dose q12 h over a 30mins infusion) while 90% of patient achieved 100%ƒT>MIC using dose (20 mg/kg q8h over 4-h infusion or 40 mg/kg q8h over 2-h infusion)	20 mg/kg dose q8h over a 4-h infusion or 40 mg/kg q8h over 2-h infusion gives optimal antibiotic coverage for susceptible pathogens

Cl: Clearance, V1: volume of distribution in central compartment, V2: volume of distribution in peripheral compartment, Vd: Volume of distribution, Cmax: maximum concentration of drug, Cmin AUC: area under curve; t1/2 = half-life MIC: minimum inhibitory concentration; T>MIC: time above minimum inhibitory concentration, TID: three times a day, BID: two times a day, OD: once daily, Ke: Elimination rate constant, PK: Pharmacokinetic, PD: Pharmacodynamic, CPB: Cardiopulmonary bypass, BSI: Blood stream infections, UTI: Urinary tract infections; PTA: Probability of target attainment, VAP: Ventilator-acquired pneumonia, CAP: Community acquired pneumonia, GIT: Gastrointestinal tract infections.

**TABLE 2:** Dose optimization of β-lactams among adults.

Antibiotics	Author and year	Country	Study design	Sample size	Characteristics of patients	Dosing practice	Pk parameter	Patients achieving targets (PAT)/Clinical outcomes	Dosing recommendation
Penicillins									
Temocillin Laterre et al. (2015)	Laterre et al. (2015)	Belgium	RCT	32	Patients with intra-abdominal and LRTIs	Loading dose: 750 mg followed by continuous infusion of 750 mg/24 h	Cl (3.69 L/h/kg); V1 (14.0 L); V2 (21.7 L); AUC0-24 (1764 mg.h/L); Cmax (170 mg/L); Cmin (51 mg/L)	A target of 80% fT.>MIC was achieved using MIC of 16 mg/L	The dosing regimen of 6 g OD by CI improve PK/PD target using a MIC of 16 mg/L
Ampicillin + sulbactam Yokoyama et al. (2015)	Yokoyama et al. (2015)	Japan	Cohort	8	Patients undergoing cardiovascular surgery with CBP	1 g/0.5 IV every 3, 4, 6 and 12 h	Vd (15.8 L); ke (0.505 h-1); half-life (1.52 h); Cl (7.72 L/h)	-	Dosing interval should be adjusted to optimize the efficacy and safety of treatment
Ampicillin + sulbactam Yokoyama et al. (2016)	Yokoyama et al. (2016)	Japan	Cohort	5	Anuric dialysis patients undergoing cardiac surgery	1 g/0.5 IV every 3, 4, 6 and 12 h	Vd (8.9 L); ke (0.18 h^−1^); half-life (4.23 h); Cl (1.69 L/h)	-	Dose should be given IV every 12 h to maintain a free drug concentration of more than 12 µg/ml
Piperacillin and tazobactum Dow et al. (2011a)	Dow et al. (2011)	Wisconsin	Retrospective cohort	129	Patients with UTIs, pulmonary, BSIs and intra-abdominal infections	Piperacillin-tazobactam infused over 4 hCrCl >20 ml/min; 3.375 g IV every 8 hCrCl <20 ml/min; 3.375 g IV every 12 hHemodialysis/peritoneal dialysis; 3.375 g IV every 12 h	-	The PTA of achieving 50% fT > MIC for prolonged infusion was 92% at MIC of 16 mg/L; 100% at MICs of <16 mg/L, while PTA was > 90% when administered 30min bolus infusion every 6 h using MIC of 1 mg/L	The utilization of prolonged infusions demonstrated the favorable outcomes
Piperacillin and Tazobactum Yost and Cappelletty, (2011)	Yost and Cappelletty, (2011)	Ohio	Retrospective cohort	359	Patients with UTIs, BSIs, RTIs and skin and soft tissues infections	4.5 g every 12 h as a 30-min infusion; 3.375 g every 8 h as a 4-h infusion; 3.375 g every 12 h as a 4-h infusion3.375 g every 12 h as a 30-min 2.25 g every 8 h as a 30-min infusion2.25 g every 12 h as a 4-h infusion	-	-	PD dosing using extended-infusion piperacillin + tazobactam improves the clinical outcomes
Piperacillin and Tazobactam Lodise et al. (2007a)	Lodise et al. (2007)	United States	Cohort	194	Patients with pseudomonas aeruginosa infections	Group 1:II (3.375 g intravenously for 30 min every 4 or 6 h)Group 2EI (3.375 g intravenously for 4 h every 8 h)	-	A 50% ∫T>MIC was achieved using dosing regimen 4-h infusion of 3.375 g of piperacillin-tazobactam administered intravenously every 8 h	The EI of drug showed to be more effective over II dosing regimen
Piperacillin Sime et al. (2015b)	Sime et al., 2007	Australia	RCT	39	Febrile neutropenic patients with hematological malignancies	4.5 g of piperacillin/tazobactam every 8 h or every 6 h		1st TDM22% patients achieved 100% ∫T>MIC and 38% patients achieved 50% ∫T>MIC.2nd TDM69% of intervention patients and 19% of control patients attained 100% ∫T>MIC, and 15/16 (94%) of intervention patients versus 5/16 (31%) of control patients achieved 50% ∫T>MIC.3rd TDM, the proportion of patients attaining 100% ∫T>MIC in 73% patients in the intervention group and 7% in the control group	TDM provides useful feedback of dosing adequacy to guide dose optimization
Piperacillin-tazobactam Roberts et al. (2010)	Roberts et al. (2010)	Australia	Cohort	16	Patients with sepsis	Piperacillin doseFor bolus 229 mg/kg/dayFor continuous 168 mg/kg/day	BolusCmax (266.6 mg/L); Cmin (7.2 mg/L); Cmin (day 2) 6.2 mg/L.ContinuousCmax (144 mg/L); Cmin (day 1) 7.1 mg/L; Cmin (day 2) 21.2 mg/L	The PTA was 93% using 16 g/day by CI and 53% using bolus dosing (4 g every 6 h)	The administration of piperacillin by CI achieved PD targets
Piperacillin Lorente et al. (2009)	Lorente et al. (2009)	Spain	cohort	87	Patients with VAP	II (4/0.5 g infused over 30min every 6 h)CI (LD 4/0.5 g over 30 min, followed by 4/0.5 g infused over 360 min every 6 h)	-	The %T>MIC was 100% for a MIC ≤ 16 mg/L for CI, the%T>MIC for II was 100% for a MIC ≤ 2 mg/L, 90% for a MIC of 4 mg/L, 70% for a MIC of 8 mg/L and 55% for a MIC of 16 mg/L	Both doses (16/2 g and 12/1.5 g) achieve serum concentrations far above the 35–40 mg/L threshold
Piperacillin De Waele et al. (2014b)	Waele et al., 2014	Belgium,	RCT	49	Patients’ pneumoniaCAP, HAP, Tracheobronchitis BSI, Peritonitis, Febrile neutropenia	LD (4 g infused over 30 min, followed by EI dose of either antibiotic (4 g PTZ) at 6-h (PTZ) dosing interval. EI doses were administered over 3 h	-	94.7% of the intervention patients achieved 100% ∫T>MIC as compared to control groups (68.4%). For the target of 100 % ∫T > 4xMIC, PTA rates were higher in the intervention group	A strategy of dose optimization based on daily TDM results in an increase in PK/PD target attainment compared to conventional dosing
Piperacillin De Waele et al. (2014a)	Waele et al., 2014	Belgium	RCT	33	Patient with normal renal functions	LD (1 g meropenem or 4 g piperacillin) administered over 30 min, followed by EI dose of either antibiotic (4 g PTZ) at 6-h (PTZ) dosing intervals. EI doses were administered over 3 h	Extended infusionCmax (76.2 mg/L); Cmin (14.7 mg/L); Cl (13.2 L/h); Vd (0.33 L/kg)Bolus infusionCmax (240.2 mg/L); Cmin (5.9 mg/L); Cl (16.2 L/h); Vd (0.36 L/kg)	Compared to bolus infusion, ∫T>MIC using extended infusion was higher for i.e. 96% compared to 77% for piperacillin	EI led to improved PK/PD target attainment
Cephalosporins									
Cefuroxime Carlier et al. (2014)	Carlier et al. (2014)	Belgium	RCT	20	Patients with pulmonary infections	II (1.5 g infused every 8 h (EI (1.5 g every 8 h or 1.5 g every 6 h)CILD 750 mg over 0.5 h constant infusion over 24 h 4.5 g over 24 h6.0 g over 24 h 7.5 g over 24 h9.0 g over 24 h	Fixed effectsCL (9.0 L/h); Vc (10.5 L); Vp(12.0 L); intercompartmental CL (18.7 L/hr)Random effects,CL (28.0 L/h); Vc (23.7 L); Vp (29.5 L)	The standard dose of 1.5 g TID leads to an 87% PTA for patients with a Cl_Cr_ of 50 ml/min and organism MIC of 8 mg/L	High-dose CI is more likely to reach PK/PD targets
Ceftazidime Cousson et al. (2015)	Cousson et al. (2015)	France	RCT	34	Patients with Ventilator-associated pneumonia	CI (LD of 20 mg/kg followed by 60 mg/kg/day)II (20 mg/kg over 30 min every 8 h)	For CIVd (0.4L/kg); AUC0-48 (1,348 mg.h/L)For IICmax (95 mg/L); Cmin (6 mg/L); V (0.3 L/kg); AUC0-48 (1,361 mg.h/L)	-	CI presentsPK/PD advantages and predictable efficacy
Ceftazidime Nicolau et al. (2001)	Nicolau et al. (2001)	United States	RCT	35	Patients with nosocomial infection	CI (3 g/day)II (2 g every 8 h)	-	-	CI presents optimal PD targets in terms of efficacy
Ceftazdime Lorente et al. (2007)	Lorente et al. (2007)	Spain	Retrospective, cohort	121	Patients with VAP	II (2 g infused over 30 min every 12 h)CI (LD of 1 g over 30 min followed by 2 g infused over 720 min every 12 h)	-	The mean time that Cp of ceftazidime increased the MIC was higher for CI (100%) than for II (99.8%, 69.0%, and 47.6% for susceptible, intermediate, and resistant strains,	Ceftazidime administered by continuous infusion had greater clinical efficacy than ceftazidime administered by intermittent infusion
Ceftazidime Buijk et al. (2002)	Buijk et al. (2002)	Netherland	RCT and non RCT	18	Patients with peritonitis	For non RCT:1 g IV loading dose followed 4.5 g IV continuous infusionFor RCT1 g IV followed by 4.5 g IV continuous infusion as above or 1.5 g IV bolus TDS for 10 days	SerumRCTAUC0-24 (1,131 mg.h/L); CL (4.1 L/h)Non-RCT:Cmax (88.7 mg/L); AUC0-24 (1,064 mg.h/L); Vd (0.279 L/h); Half-life (4.2 h); CL (5.1 L/h)	CI resulted in mean serum concentration >40 mg/L and a T4xMIC for most pathogens encountered in severe IAIs for >90% of the course of the therapying both serum and peritoneal exudate	CI resulted in more favorable concentration in serum and peritoneal exudate
Ceftazidime Hanes et al. (2000)	Hanes et al. (2000)	United States	Cohort	31	Patients with nosocomial pneumonia	2 g intravenously every 8 hours2 g an intravenous bolus followed by 60 mg/kg per day as a continuous intravenous infusion	For continuous ceftazidimeCss (19.2 mg/ml); Cl (2.45 ± 0.76 L/h) for intermittentCmax (44.3 mg/ml); Cmin (3.7 6 mg/ml); V (0.32 + 0.14 L); Half-life (1.72 + 0.71 h); Cl (2.33 + 1.06 L/h)	Both the CI and II dosing regimen maintained drug concentrations above the MIC 100% of the dosing interval in all patients	Both II and CI dosing regimens were equally effective to treat nosocomial pneumonia
Cefepime Chapuis et al. (2010)	Chapuis et al. (2010)	Switzerland	Cohort	91	Patients with mixed conditions	2 g every 12 h for Cl_Cr_ ≥ 50 ml/min IV2 g every 24 h or 36 h for Cl_Cr_ < 50 ml/min IV	1st doseCmax (105 + 22 mg/L); Cmin (7.6 + 2 mg/L); V (0.513 + 0.180 L/kg); Vss (0.413 + 0.118 L/kg); AUC (370 + 360 mg.h/L); Half-life (4.03 + 3.19 h)Steady doseCmax (97 + 8 mg/L); Cmin (2.68 + 3.06 mg/L); Vb (0.513 + 0.80 L/kg); Vss (0.413 + 0.118 L/kg); AUC (226 + 107 mg.h/L); Half-life (4.33 + 4.32 h)	All study population had appropriate duration of cefepime concentrations above the MIC (T>MIC≥50%) for the pathogens recovered (MIC ≤ 4 mg/l), but only 45–65% of them had appropriate coverage for potential pathogens using MIC ≥ 8 mg/L	The dose of 2 g every 12 h provides the safety and efficacy window in patients with a Cl_Cr_ ≥ 50 ml/min infected by pathogens with cefepime MICs ≤ 4 mg/l
Ceftriaxone Joynt et al. (2001)	Joynt et al. (2001)	Hong Kong	Cohort	12	Patients with pneumonia, septic shock, sepsis, bacteremia	2 g OD as an infusion over 30 min	Cmax (204.9 mg/L); Vc (5.9 L); Vss (19.9 L); Cl (41.3 ml/min);	-	Decrease in dosing interval or CI should be evaluated further in patients with normal renal function
Ceftriaxone Roberts et al. (2007)	Jason et al., 2007	Australia	RCT	57	Patients with sepsis	2 g administered once a day as a bolus2 g as a 24 h infusion	-	-	Improvement in the primary endpoints in terms of efficacy was observed for patients receiving CI for 4 or more days
Ceftriaxone And Cefepime (Lodise et al., 2007b)	Lodise et al. (2007)	Germany	Cohort	14	Patients with extracerebral infections	Ceftriaxone 2 g IV q12 h and cefepime 2 g IV q8h	-	For ceftriaxone, The PTA of achieving 50% and 100% ∫T>MIC in the CSF were 76% and 65% respectively. For cefepime, the PTA at 50% and 100% ∫T>MIC in the CSF were 91.8% and 82%, respectively	The CSF PD against S. pneumoniae for cefepime were superior to that of ceftriaxone
Cefpirome Kang et al. (2020)	Kang et al. (2020)	Republic of Korea	cohort	15	Patients receiving Extracorporeal oxygenation	2 g cefpirome every 12 h (q12 h) as an intravenous bolus injection	Based model population estimateCl (3.6 L/h); Vc (10.3 L); Vp (19.5 L)		2 g cefpirome q8h (6 g/day) for IV bolus or 2 g every 12 h for EI over 4 h is recommended
Carbapenems									
Meropenem Dow et al. (2011b)	Rebekka et al., 2011	United States	Cohort, Retrospective	121	Patients with UTIs, pulmonary, BSIs and intra-abdominal infections	Meropenem infused over 3 hCl_Cr_ > 36 ml/min (500 mg IV every 6 h); Cl_Cr_ 26–35 ml/min (500 mg IV every 8 h); Cl_cr_ 10–25 ml/min (500 mg IV every 12 h); Cl_Cr_ 10 ml/min (500 mg IV every 24 h); Hemodialysis/peritoneal dialysis (500 mg IV every 24 h)	-	The mean drug exposures (%∫T>MIC) above the MICs of 4 and 1 mg/L of 47.27% and 71.44% of the dosage interval	The prolonged infusions showed to be effective and improve clinical outcomes in critically ill patients
Meropenem Yokoyama et al. (2018)	Yokoyama et al. (2018)	Japan	Cohort	4	Patients receiving hemodiafiltration	0.5 g OD (1 h infusion)	Vd (15.80 L); CLnon-I-HDF (1.05 ± 0.27 L/h); CLI-HDF (5.78 ± 1.03 L/h)	Dosing regimens of 0.25 g OD for a MIC of 8 mg/ml and of 0.5 g once daily for a MIC of 16 mg/ml achieved 40% T > MIC.	0.5 g OD is considered an appropriate regimen for empirical treatment
Meropenem Crandon et al. (2011)	Crandon et al. (2011)	United States	Cohort	21	Patient with VAP	0.5 g q6h (0.5 h inf)1 g q8h (0.5 h inf)2 g q8h (0.5 h inf)2 g q8h (3 h inf)	-	At MICs up to 8 mg/L, the PTA using 40% fT > MIC was 96%, 90%, and 61% for 3 h infusions of 2 g q8h, 1 g q8h, and 1 g q12 h in patients with Clcr ≥50, 30–49, and 10–29	Meropenem doses of 2 g every 8 h (3 h infusion) were required to achieve predictable PTA against MICs ≤8 μg/ml
Meropenem Lorente et al. (2006)	Lorente et al. (2006)	Spain	Cohort	89	Patient with VAP	CI (1 g over 360 min every 6 h)II (1 g over 30 min every 6 h)	-	The group receiving CI showed greater clinical rate (90.47%) than another group receiving II (59.57%)	CI may have more clinical efficacy in the treatment of VAP.
Meropenem Lu et al. (2016)	Lu et al. (2016)	China	Cohort	42	Patients with post neurosurgery, meningitis	1 g every 8 h (q8h)1 g q6h2 g q8h	Clc (22.2 L/h); Clp (1.79 L/h); Vc (17.9 L); Vp (3.84 L)	A 4-h infusion with a limited CSF drainage rate has a >90% probability of achieving 40% *T*>MIC for MICs of ≤8 mg/L. In CSF, it had a >90% PTA of achieving 50% and 100% *T*>MIC for MICs of ≤ 0.5 mg/L and ≤ 0.25 mg/L, and has a >80% PTA of achieving 50% and 100% *T*>MIC for MICs of ≤1 mg/L and ≤0.5 mg/L	2 g every 8 h, administered as a 4-h infusion with a limited CSF drainage rate (less than 150 ml/day), may provide the highest possibility of target attainment
Meropenem Kothekar et al. (2020)	Kothekar et al. (2020)	India	Cohort	25	Patients with severe sepsis and septic shock	1,000 mg as a 3 h Extended Infusion (Q8H)	Day 1Cmax (15.36 µg/ml); AUC (57.92 μg.h/ml); Half-life (1.31 h); Cl (17.26 L/h); Vd (32.61 L)Day 3Cmax (14.14ug/ml); AUC (43.82ug.hr/ml); Half-life (0.6 h); Cl (22.86 L/h); Vd: (19.83 L)	100% patients achieved targets of 40%fT > MIC while 66.7% patients achieved targets of 40%fT > 2×MIC	It requires a bolus of 500 mg followed by EI of 1,500 mg Q8H. While fT > 8 µg/ml > 40 require escalation of EI dose, fT > 4 µg/ml = 100 and fT > 8 µg/ml = 100 require escalation of both EI dose and frequency
Meropenem Cheatham et al. (2008)	Cheatham et al. (2008)	Indiana	Prospective, open-label, steady-state pharmacokinetic study	20	Patients with bacterial infection	30-min infusions of meropenem500 mg every 6 h (group 1) every 8 h (group 2)every 12 h (group 3)	Group 1Cmax (29.2 µg/ml); Cmin (2.4 µg/ml); Half-life (2.5 h); Cl (10.7 L/h); AUC (49.1 μg.h/ml); V (29.3 L)Group 2Cmax (33.2 µg/ml); Cmin (3.8 µg/ml); Half-life (3.4 h); Cl (6.4 L/hr); AUC (86.2 µg-h/ml); V (23.8 L)Group 3Cmax (33.5 µg/ml); Cmin (4.9 µg/ml); Half-life (6.1 h); Cl (3.7 L/hr); AUC (140.2ug.h/ml); V: (0.38 L)	At 40% ∫T>MIC, the PTA was 90.2%, 95.6%, and 99.5% for groups 1, 2, and 3, respectively	PD analysis suggest that regimens of meropenem 500 mg every 6, 8, or 12 h, adjusted for renal function, are sustainable for treatment of infectious diseases
Meropenem De Waele et al. (2014a)	Waele et al., 2014	Belgium	RCT	33	Patient with renal function	LD 1 g followed EI dose of 1 g every 8 hoursEI doses are administered over 3 h	Extended infusionCmax (17 mg/L); Cmin (14.7 mg/L); Cl (15.9 L/hr); Vd (0.39 L/kg)Bolus infusionCmax (85.2 mg/L); Cl (15.7 h^−1^); Vd (0.24 L/kg)	Compared to bolus infusion, ∫T>MIC using EI was higher for 82% compared to 51%	EI led to improved PK/PD target attainment
Imipenem Sakka et al. (2007)	Sakka et al. (2007)	Germany	RCT	20	Patients with nosocomial pneumonia	LD of 1 g/1 g imipenem and cilastatin (as a short-term infusion) followed by 2 g/2 g imipenem-cilastatin per 24 h as a CI for 3 days	-	II of 1 g q8h had a 90% PTA for achieving fT_MIC of 20% at MIC of 8 mg/L, while this was 4 mg/L for the fT_MIC target of 30% and 1–2 mg/L for the fT_MIC target of 40% (88% probability at 2 mg/L). For CI, all three targets were achieved at the 90% probability level at an MIC of 2–4 mg/L (86% at 4 mg/L)	It provides robust coverage for the most common nosocomial pathogens when administered either in II or CI.
Doripenem Hsaiky et al. (2013)	Hsaikay et al. (2013)	United States	Cohort study	200	Patients with pneumonia, SSTIs, UTIs, intraabdominal infections	Cl_cr_ > 50 ml/min (500 mg every 8 h).Cl_cr_ 30 ml/min or more to 50 ml/min or less (250 mg every 8 h)Cl_cr_ l 30–10 ml/min (250 mg every 12 h)Cl_cr_ less than 10 ml/min (500 mg after hemodialysis)	-	-	Doripenem should be administered via prolonged infusion when required
Aztreonem Cies et al. (2017a)	Cies et al. (2017)	United States	Case report	1	Patient with injury, chronic respiratory failure, and a tracheostomy	2 g IV every 6 h (each dose infused over 4 h) and polymyxin B 1,000,000 units IV every 12 h (each dose infused over 30 min) on 3rd day		The PTA of 100% for serum and presumed ELF concentration above the MIC for at least 40% of the dosing interval	A prolonged infusion regimen of aztreonam 2 g every 6 h (each dose infused over 4 h) was effective in this complex patient with MDR P aeruginosa empyema

Cl: Clearance, V1: volume of distribution in central compartment, V2: volume of distribution in peripheral compartment, Vd: Volume of distribution, Cmax: maximum concentration of drug, Cmin AUC: Area under curve; t1/2 = half-life MIC: Minimum inhibitory concentration; LD: Loading dose, TID: three times a day, BID: Two times a day, OD: once daily, Ke: Elimination rate constant, PK: Pharmacokinetic, PD: Pharmacodynamic, CPB: Cardiopulmonary bypass, BSI: Blood stream infections, UTI: Urinary tract infections; PTA: Probability of target attainment, VAP: Ventilator-acquired pneumonia, CAP: Community acquired pneumonia, GIT: Gastrointestinal tract infections, LRTIs: Lower respiratory tract infections; MDR: Multi-drug resistant; Clcr: Creatinine clearance; SSTIs: Skin and Soft tissue infections, HAP: Hospital-acquired pneumonia, TDM: Therapeutic drug monitoring, RCT: Randomized Controlled Trials, CI: continuous infusion, II: Intermittent infusion, EI: Extended infusion.

### Dose optimization of β-lactams in pediatrics

A total of twenty studies were reported among pediatrics (Table 1). Of 20 studies, eight studies were reported on penicillins (Cies et al., 2014; De Cock et al., 2015; Nichols et al., 2016; De Cock et al., 2017; Béranger et al., 2019; Tang et al., 2019; D’Agate et al., 2020; Wu et al., 2021), eight on cephalosporins (Olguin et al., 2008; Fukumoto et al., 2009; De Cock et al., 2016; Cies et al., 2018a; Béranger et al., 2018; Cies et al., 2019; Hartman et al., 2019) and 4 on carbapenems (Giannoni et al., 2006; Cies et al., 2015; Cies et al., 2017b; Tan et al., 2018) (Table 1). Four studies were reported on amoxicillin (De Cock et al., 2015; Tang et al., 2019; D’Agate et al., 2020; Wu et al., 2021). The normal dose for amoxicillin in selected studies ranged from 25 mg/kg to 125 mg/kg. Wu and his colleagues recommended to use other broad-spectrum antibiotic instead of amoxicillin for the treatment of *E. coli* infections. Another study reported that administration of dose (25 mg/kg every 6 h) of amoxicillin + clavulanic acid was stopped due to clinical failure in critically ill pediatrics with augmented renal functions (De Cock et al., 2015). The dose optimization and PK/PD parameters of piperacillin/tazobactam were discussed in four cohort studies (Cies et al., 2014; Nichols et al., 2016; De Cock et al., 2017; Béranger et al., 2019). The recommended dose of piperacillin range was from 150 mg/kg to 450 mg/kg. The continuous or extended infusion of piperacillin was shown to be effective in terms of safety and efficacy. De Cock et al. reported that loading dose followed by continuous infusion may improve the PD targets (De Cock et al., 2017).

Two cohort studies on cefazolin were reported in pediatrics (De Cock et al., 2016; Cies et al., 2019). The authors proposed a dose of 25 mg/kg by assessing the PK parameters using the Monta Simulation Model. The authors recommended that mixing cefazolin in the CPB circuit priming solution was effective in maintaining cefazolin serum concentration during surgery (De Cock et al., 2016). Two cefotaxime studies were included in this review (Béranger et al., 2018; Hartman et al., 2019). The recommended dose of cefotaxime ranges from 100 mg to 300 mg/kg as a continuous infusion that achieved 100% probability target attainment (PTA). Olguin et al. (2008) studied the PK parameters of cefuroxime on 11 patients with septicemia and septic shock. The authors recommended the dose of 100 mg/kg of body weight, administered every 6 h by intravenous infusion for 30 min. Cies et al. (2018a) discussed the PK-PD characteristics of Ceftaroline on 7 patients with MRSA infection. In this study, majority of the patients did not require additional alteration to achieve target attainment while a dose of 15 mg/kg was recommended for patients with increased volume of distribution.

One cohort study was reported on imipenem (Giannoni et al., 2006). All patients using dose regimen 100 mg/kg/day reached *∫*T>MIC of 70%–100% for all isolated pathogens except methicillin-resistant *staphylococcus* epidermidis pathogen. Three studies were found reporting meropenem using the same dose (40 mg/kg) in pediatrics (Cies et al., 2015; Cies et al., 2017b). However, these studies recommended the continuous dosing regimen resulted in effective therapy.

### Dose optimization of β-lactams in adults

A total of 32 studies were reported in adults, of which 11 articles were on penicillins (Lodise et al., 2007a; Lorente et al., 2009; Roberts et al., 2010; Dow et al., 2011b; Yost and Cappelletty, 2011; De Waele J. et al., 2014; De Waele J. J. et al., 2014; Sime F. B. et al., 2015; Laterre et al., 2015; Yokoyama et al., 2015; Yokoyama et al., 2016), 11 on cephalosporin (Hanes et al., 2000; Joynt et al., 2001; Nicolau et al., 2001; Buijk et al., 2002; Lodise et al., 2007b; Lorente et al., 2007; Roberts et al., 2007; Chapuis et al., 2010; Carlier et al., 2014; Cousson et al., 2015; Kang et al., 2020), 10 on carbapenems (Lorente et al., 2006; Sakka et al., 2007; Cheatham et al., 2008; Dow et al., 2011a; Crandon et al., 2011; Hsaiky et al., 2013; De Waele J. J. et al., 2014; Lu et al., 2016; Yokoyama et al., 2018; Kothekar et al., 2020) and 1 on other β-lactams (aztreonam) (Cies et al., 2017a) (Table 2). One randomized controlled trial was conducted on temocillin in patients with intra-abdominal and lower respiratory tract infections (Laterre et al., 2015). A target of 80% *∫*T>MIC was reached for the mean population for a MIC of 16 mg/L and a target of around 40 was reached for the mean population for a MIC of 32 mg/L. Two cohort studies were performed on patients receiving ampicillin + sulbactam (Yokoyama et al., 2015; Yokoyama et al., 2016). The standard dose of 1 g/0.5 g intravenously seemed to be adequate in terms of efficacy. However, dosing intervals can be increased to optimize the safety and efficacy of the treatment. Six studies were documented on piperacillin with or without combination with tazobactam, out of which two are randomized controlled trials (RCT) (Lodise et al., 2007a; Lorente et al., 2009; Roberts et al., 2010; Dow et al., 2011b; Yost and Cappelletty, 2011; Sime F. B. et al., 2015). Most of the studies recommended the dose of piperacillin of 4.5 g every 6 h or 8 h infused over 30 min. The administration of piperacillin + tazobactam using extended or continuous infusion achieve superior PK/PD targets.

For cefuroxime, one RCT was reported (Carlier et al., 2014). The standard dose of cefuroxime prescribed by physicians was 1.5 g TID. Carrier et al. recommended that high-dose continuous infusion is more likely to reach PK/PD targets. The standard dose leads to 87% probability of target attainment (PTA) for patients with creatinine clearance (CLCr) of 50 ml/min and pathogen of MIC 8 mg/ml. Five studies were reported on ceftazidime (Hanes et al., 2000; Nicolau et al., 2001; Buijk et al., 2002; Lorente et al., 2007; Cousson et al., 2015). All these studies recommended the continuous infusion regimen that presents PK/PD advantages and predictable efficacy. Lorente et al. (2007) reported that the meantime that plasma ceftazidime concentration exceeded the MIC was higher for continuous infusion (100%) for susceptible, intermediate and resistant strains over intermittent infusion. Chapuis and his colleagues studied the PK/PD parameters of cefepime which identified a safety and efficacy window for a dose of 2 g every 12 h in patients with ClCr > 50 ml/min infected by pathogens with cefepime < 4 mg/ml. The dose of ceftriaxone included in three studies was 2 g once daily (Chapuis et al., 2010).

Eight studies were reported on dose optimization and PK/PD characteristics of meropenem (Lorente et al., 2006; Cheatham et al., 2008; Dow et al., 2011b; Crandon et al., 2011; De Waele J. J. et al., 2014; Lu et al., 2016; Yokoyama et al., 2018; Kothekar et al., 2020). In Cheathman et al. (2008) study, the PK/PD analysis recommended that dosing regimen of meropenem 500 mg every 6, 8, or 12 h, adjusted for the renal function is considered for treatment of various infection. Similarly, Kothekar et al. reported that dose optimization of meropenem is required in patients with severe sepsis and septic shock. The prescribed was 100 mg as 3 h extended infusion. The PTA was 100% at 40% *∫*T>MIC and 66.7% at 40% *∫*T > 2xMIC. Sakka et al. (2007) reported that imipenem-colistin provide robust coverage for most common nosocomial pathogens when administered either in intermittent or continuous infusion of 1 g q8h or in a continuous infusion of 2 g/day. Hsaikay et al. (2013) reported that doripenem should be administered via prolonged infusion regimen to optimize the efficacy of the treatment The dose of aztreonam 2 g every 6 h was effective in patients with pseudomonas aeruginosa empyema (Cies et al., 2017a).

### Quality assessment

In NOS, a maximum of 13 stars assigned to each study. According to Agency for Healthcare Research and Quality (AHRQ) standards, a study who scored 3 or 5 stars in selection, 1 or 2 stars in comparability group and 2 or 3 stars in outcome groups is of good quality, study who scored 2 stars in selection domain, 1 or 2 stars in comparability domain and 2 or 3 stars in outcome domain is of fair quality, study who scored 0 or 1 start in selection group of 0 stars in comparability group or 0 or 1 star in outcome group is of poor quality. In this systematic review, out of 52 studies, 50 studies are of good quality and the remaining two studies are of fair quality (Table 3). The Cochrane bias tool assessed that all RCT studies are at lower risk of bias and all domains were discussed in Table 4. The two case reports included in the systematic review are of good quality (Table 5).

**TABLE 3:** Quality assessment of cohort studies.

References	Selection				Comparability	Outcomes		
	Representative of exposed studies^a^	Selection of non-exposed^b^	Ascertainment of exposure^c^	Demonstration of outcome^d^	Comparability of cohort studies on basis of design^e^	Assessment of outcomes^f^	Adequacy of follow-up^g^	Quality score
Tang et al. (2019) Tang et al. (2019)	*	*	*	*	*	*	*	7
Wu et al. (2021) Wu et al. (2021)	*	*	*	*	*	*	*	7
D’Agate et al. (2020) D’Agate et al. (2020)	*	*	*	*	*	*	*	7
De Cock et al. (2015) De Cock et al. (2015)	*	*	*	*	*	*	*	7
Agathe Béranger et al. (2019) Béranger et al. (2019)	*	*	*	*	*	*	*	7
Cies et al. (2014) Cies et al. (2014)	*	*	*	*	*	*	*	7
Nichols et al. (2016) Nichols et al. (2016)	*	*	*	*	*	*	*	7
De Cock et al. (2017) De Cock et al. (2017)	*	*	*	*	*	*	*	7
Cies et al. (2019) Cies et al. (2019)	*	*	*	*	*	*	*	7
De Cock et al. (2016) De Cock et al. (2016)	*	*	*	*	*	*	*	7
a.Béranger et al., 2018 Béranger et al. (2018)	*	*	*	*	*	*	*	7
Olguin et al. (2008) Olguin et al. (2008)	*	*	*	-	*	*	*	6
Fukumoto et al. (2009) Fukumoto et al. (2009)	*	*	*	-	*	*	*	6
Khan et al. (2020) Khan et al. (2020)	*	*	*	*	*	*	*	7
Giannoni et al. (2006) Giannoni et al. (2006)	*	*	*	*	*	*	*	7
Tan et al. (2018) Tan et al. (2018)	*	*	*	*	*	*	*	7
Yokoyama et al. (2015) Yokoyama et al. (2015)	*	*	*	-	-	**	*	6
Yokoyama et al. (2016) Yokoyama et al. (2016)	*	*	*	-	-	**	*	6
Dow et al. (2011) Dow et al. (2011a)	*	*	*	*	*	**	*	8
Yost & Cappelletty, (2011) Yost and Cappelletty, (2011)	*	*	*	-	*	**	*	7
Lodise et al. (2007) Lodise et al. (2007a)	*	*	*	*	*	**	*	8
J. A. Roberts et al., 2010 Roberts et al. (2010)	*	*	*	*	*	*	*	7
Lorente et al. (2009) Lorente et al. (2009)	*	*	*	*	*	**	*	8
Lorente et al. (2007) Lorente et al. (2007)	*	*	*	*	*	**	*	8
Buijk et al. (2002) Buijk et al. (2002)	*	*	*	*	*	*	*	7
Hanes et al. (2000) Hanes et al. (2000)	*	*	*	*	*	*	*	7
Chapuis et al. (2010) Chapuis et al. (2010)	*	*	*	*	*	*	*	7
Joynt et al. (2001) Joynt et al. (2001)	*	*	*	*	*	*	*	7
Lodise et al. (2007) Lodise et al. (2007b)	*	*	*	*	*	*	*	7
Kang et al. (2020) Kang et al. (2020)	*	*	*	*	*	*	*	7
Yokoyama et al. (2018) Yokoyama et al. (2018)	*	*	*	*	*	**	*	8
Crandon et al. (2011) Crandon et al. (2011)	*	*	*	*	*	*	*	7
Lorente et al. (2006) Lorente et al. (2006)	*	*	*	*	*	**	*	8
Lu et al. (2016) Lu et al. (2016)	*	*	*	*	*	*	*	7
Kothekar et al. (2020) Kothekar et al. (2020)	*	*	*	*	*	*	*	7
Cheatham et al. (2008) Cheatham et al. (2008)	*	*	*	*	*	*	*	7
Hsaiky et al. (2013) Hsaiky et al. (2013)	*	*	*	*	*	*	*	7
Cies et al. (2017) Cies et al. (2017a)	*	*	*	*	*	*	*	7
Cies et al. (2018) Cies et al. (2018a)	*	*	*	*	*	**	*	8

a : * = truly representative or somewhat representative of average in target population.

b : * = Drawn from the same community.

c : * = Secured record or structured review.

d : * = Yes, - = No.

e : * = Study controls for age, gender, and other factors.

f : * = Record linkage or blind assessment, ** = Both.

g : * = follow-up of all subjects.

**TABLE 4:** Risk of bias assessment for randomized controlled trials.

Study	Random sequence generation	Allocation concealment	Blinding of participants and personnel	Blinding of outcome assessment	Incomplete outcome data	Selective reporting	Other bias
Hartman et al. (2019)	Low risk	Low risk	High risk	High risk	Unclear	Unclear	Unclear
Laterre et al. (2015)	Low risk	Low risk	High risk	High risk	Unclear	Low risk	Unclear
Sime et al. (2015b)	Low risk	Low risk	High risk	High risk	Low risk	Low risk	Unclear
De Waele et al. (2014b)	Low risk	Low risk	High risk	High risk	Low risk	Low risk	Unclear
De Waele et al. (2014a)	Low risk	Low risk	High risk	High risk	Low risk	Low risk	Unclear
Carlier et al. (2014)	Low risk	Low risk	High risk	High risk	Low risk	Low risk	Unclear
Cousson et al. (2015)	Low risk	Low risk	Unclear	High risk	Low risk	Low risk	Unclear
Nicolau et al. (2001)	Low risk	Low risk	Unclear	High risk	Unclear	Low risk	Unclear
Roberts et al. (2007)	Low risk	Low risk	High risk	High risk	Low risk	Low risk	Unclear
Sakka et al. (2007)	Low risk	Low risk	High risk	High risk	Low risk	Low risk	Unclear

**TABLE 5:** Quality assessment of case reports.

Study	Q1	Q2	Q3	Q4	Q5	Q6	Q7	Q8	Quality rating
Cies et al. (2015) Cies et al. (2015)	Yes	Yes	Yes	No	Yes	Yes	No	Yes	Good
Cies et al. (2017) Cies et al. (2017a)	Yes	Yes	Yes	No	Yes	Yes	No	Yes	Good

Q1. Were patient’s demographic characteristics clearly described?

Q2. Was the patient’s history clearly described and presented as a timeline?

Q3. Was the current clinical condition of the patient on presentation clearly described?

Q4. Were diagnostic tests or assessment methods and the results clearly described?

Q5. Was the intervention(s) or treatment procedure(s) clearly described?

Q6. Was the post-intervention clinical condition clearly described?

Q7. Were adverse events (harms) or unanticipated events identified and described?

Q8. Does the case report provide takeaway lessons?

## Discussion

Inappropriate antibiotic treatment is most often the result of inappropriate dose, delayed administration or more often an underestimation of current trends in resistance (Sulis et al., 2020). The bactericidal activity of antibiotics depends on the concentration of the drug with regards to the minimum inhibitory concentration (MIC) and the time that this exposure can be sustained (Kuti, 2016). The MIC represents the most fundamental PD measure for antibiotics against pathogens, presenting the potency of administered antibiotics (Onufrak et al., 2016). The dose optimization based on MIC would seem to provide rectification in the PD characteristics and target attainment (Hartman et al., 2020). However, the demerits using MIC values to optimize the dosing regimens were highlighted by Mouton et al. (2018). Therefore, MIC variation must be examined to avoid potential underdosing of the patient. Moreover, alteration in PK measure may affect the PD characteristics. In our systematic review, we have gathered information regarding the dosing pattern of β-lactams from 52 studies. The majority of the studies were carried out in intensive care units. Although antibiotic use is the cornerstone of intensive care treatment for critically ill patients with suspected infection (Pickens and Wunderink, 2019).

β-lactams include penicillins, cephalosporins, and carbapenems are widely used in the management and treatment of serious infection particularly in critically ill patients (Thakuria et al., 2013; Bozcal et al., 2017). All β-lactams showed time-dependent bactericidal activity, which is determined by the free antibiotic concentration-time above the MIC for microorganisms identified (%*∫*T>MIC) (Masich et al., 2018a; Pandey and Cascella, 2020). The optimal clinical outcomes may differ depending on the β-lactams, for example, the target attainment goals for piperacillin + tazobactam, cephalosporins and carbapenems were 50%*∫*T>MIC, 60%*∫*T>MIC and 40%*∫*T>, respectively (Masich et al., 2018a). Moreover, the maximal bactericidal activity can be achieved by increasing the drug levels i.e., four to five times above MIC, even so, the interaction to improved clinical outcomes is inconsistent. The specific percentage of dosing interval *∫*T>MIC needed for optimal activity differs for different β-lactam classes. The variation in percentages have been associated with variation in the rate of killing and the post-antibiotic effect. Majority of the studies documented the clinical pharmacodynamic parameters of β-lactams against gram-negative bacteria (Manduru et al., 1997; Tam et al., 2002). Several studies suggested that the amount of time the plasma concentration of the drug remains 4-6 fold greater than MIC has to be maintained for 100% of the dosing time period, however other studies have reported a target of 60% *∫*T>MIC depending on clinical outcome measures (clinical cure vs. reduced bacterial resistance) (Manduru et al., 1997; Tam et al., 2002; Crandon et al., 2010).

As per available evidence, the current knowledge of PK and target attainment is often suboptimal in patients following the standard dosing regimen of β-lactams. Most of the studies provide data on PK parameters (34/52). It is evident that changes in PK parameters occur in patients. Cies et al. (2018a) reported that volume of distribution was increased in 86% of patients and clearance was increased in 71% of patients receiving ceftaroline. Piperacillin (12/52) was the most commonly used β-lactams, followed by meropenem (11/52), and ceftazidime (5/52). Piperacillin and meropenem are widely used to treat various infections among the hospitalized patients because of their susceptibility against many gram-positive and gram-negative pathogens (Shah and Ryzner, 2013; Xu et al., 2019).

The common mode of administration of antibiotics recommended by many clinicians was intermittent intravenous administration (Kasiakou et al., 2005). However, optimal dosing strategies for the treatment of various infectious diseases remain controversial. Most of the β-lactams were administered as an intermittent bolus. However, on the basis of strong PK/PD data, the administration of antibiotics by continuous infusion is more effective than administration by intermittent infusion (Dulhunty et al., 2013). Many of included studies found that continuous or extended infusion increased the survival rates among hospitalized patients especially critically ill patients. The administration of β-lactams as continuous infusion increased blood and interstitial fluid concentration with greater time above the MIC as compared to intermittent dosing, especially for pathogens with MIC values, which are frequent in ICUs (Dulhunty et al., 2013). The potential benefits to patients as well as the healthcare system by implementing improved approaches of antibiotic delivery are substantial. In an era of increasingly expensive treatments, the administration of β-lactams are cost-effective in terms of drug costs and labor costs (Mouton and Vinks, 2007).

β-l actams are frequently recommended by international and national treatment guidelines, have been prescribed for various infectious diseases. Therefore, ASPs should be implemented that helps the clinicians to use antibiotic appropriately from a pharmacological point of view that means excluding the pharmacological factors that potentially increase the risk of spread of resistance (Adembri et al., 2020). More accurately, antibiotics should be administered following PK/PD principles. When selecting the appropriate dosing regimen by keeping in view the PK/PD principles, the specified pathophysiological changes must be taken into consideration (Roberts et al., 2014). Moreover, multiple PK/PD software using a combination of TDM, Bayesian forecasting and PopPK can be utilized by pharmacists, clinical pharmacologists, and clinicians to maintain optimal target attainment (Abdulla et al., 2021). The guidelines on the use of TDM including an overview of suggested PD targets for several B-lactams antibiotics is also recommended by the French Society Anesthesia and Intensive Care Medicine (SFAR) (Guilhaumou et al., 2019). However, various softwares such as MIPD, NONMEM, MWPHARM++, ID-ODS, InsightRx Nova and AutoKinetics are available, close collaboration between pharmacists and clinicians are required to implement this feature to optimize the patient target attainment (Sime F. et al., 2015; Kantasiripitak et al., 2020). Model-informed precision dosing (MIPD) is an emerging approach that improves TDM process. This approach estimates the PK variability utilizing population PK model and predict the probability of target attainment for various dosing regimen (Gijsen et al., 2022). Despite of its advantages and availability of softwares, adoption of MIPD in clinical settings has been limited to date (Neely et al., 2018; Frymoyer et al., 2020).

The present study has some limitation that should be acknowledged when evaluating the data from included studies. Firstly, this study used limited databases with specific focus on titles describing the dose optimization of β-lactams antibiotics as no quantitative analysis was carried out. Moreover, limited grey literature search was conducted using additional search terms that identified relevant data. Secondly, some studies included the co-administration of two or more β-lactams antibiotics may alter the PK/PD parameters of both drugs. Thirdly, the difficulty in the assessment of efficacy concerning MIC was observed due to under-reporting.

## Conclusion

This systematic review showed that appropriate antibiotic therapy is challenging due to a wide range of pathophysiological change among different age groups. This challenging perspective requires close collaboration between clinicians, pharmacists and clinical pharmacologists to optimize the effective treatment and improve the clinical outcome. The PK/PD analysis can be utilized to support alternative dosing regimens such as increase in dosing interval, continuous infusion, and increased bolus doses. The current study aimed to inspire both researchers and clinicians to identify and resolve these differences, not only by elucidating PK/PD parameters, but also providing guidelines for implementation in the healthcare settings, as this data is important to optimize antibiotic treatment in patient populations.

## Data Availability

The original contributions presented in the study are included in the article/Supplementary Material, further inquiries can be directed to the corresponding author.
